# Antitumor Activity against Human Colorectal Adenocarcinoma of Silver Nanoparticles: Influence of [Ag]/[PVP] Ratio

**DOI:** 10.3390/pharmaceutics13071000

**Published:** 2021-07-01

**Authors:** Omar Ulises Cruz-Ramírez, Lucía Margarita Valenzuela-Salas, Alberto Blanco-Salazar, José Antonio Rodríguez-Arenas, Paris A. Mier-Maldonado, Juan Carlos García-Ramos, Nina Bogdanchikova, Alexey Pestryakov, Yanis Toledano-Magaña

**Affiliations:** 1 Centro de Nanociencias y Nanotecnología, Universidad Nacional Autónoma de México, Ensenada 22860, Mexico; omarulisescruzramirez@gmail.com (O.U.C.-R.); nina@cnyn.unam.mx (N.B.); 2 Facultad de Ciencias de la Salud Unidad Valle de las Palmas, Universidad Autónoma de Baja California, Tijuana 22260, Mexico; lucia.valenzuela@uabc.edu.mx (L.M.V.-S.); paris.mier@uabc.edu.mx (P.A.M.-M.); 3 Programa de Maestría y Doctorado en Ciencias e Ingeniería, Facultad de Ciencias, Universidad Autónoma de Baja California, Ensenada 22860, Mexico; alberto.blanco.salazar@uabc.edu.mx (A.B.-S.); jose.antonio.rodriguez.arenas@uabc.edu.mx (J.A.R.-A.); 4 Escuela de Ciencias de la Salud Unidad Valle Dorado, Universidad Autónoma de Baja California, Ensenada 22890, Mexico; 5 Research School of Chemistry & Applied Biomedical Sciences, National Research Tomsk Polytechnic University, 634050 Tomsk, Russia; pestryakov2005@yandex.ru

**Keywords:** AgNPs, antitumor activity, colon cancer, HCT-15, cytotoxic selectivity, [metal]/[coating agent] ratio, therapeutic index, GSH classification

## Abstract

Silver nanoparticles (AgNPs) not only have shown remarkable results as antimicrobial and antiviral agents but also as antitumor agents. This work reports the complete characterization of five polyvinylpyrrolidone-coated AgNP (PVP-AgNP) formulations, their cytotoxic activity against human colon tumor cells (HCT-15), their cytotoxic effect on primary mouse cultures, and their lethal dose on BALB/c mice. The evaluated AgNP formulations have a composition within the ranges Ag: 1.14–1.32% *w*/*w*, PVP: 19.6–24.5% and H_2_O: 74.2–79.2% with predominant spherical shape within an average size range of 16–30 nm according to transmission electron microscopy (TEM). All formulations assessed increase mitochondrial ROS concentration and induce apoptosis as the leading death pathway on HCT-15 cells. Except for AgNP1, the growth inhibition potency of AgNP formulations of human colon tumor cancer cells (HCT-15) is 34.5 times higher than carboplatin, one of the first-line chemotherapy agents. Nevertheless, 5–10% of necrotic events, even at the lower concentration evaluated, were observed. The cytotoxic selectivity was confirmed by evaluating the cytotoxic effect on aorta, spleen, heart, liver, and kidney primary cultures from BALB/c mice. Despite the cytotoxic effects observed in vitro, the lethal dose and histopathological analysis showed the low toxicity of these formulations (all of them on Category 4 of the Globally Harmonized System of Classification and Labelling of Chemicals) and minor damage observed on analyzed organs. The results provide an additional example of the rational design of safety nanomaterials with antitumor potency and urge further experiments to complete the preclinical studies for these AgNP formulations.

## 1. Introduction

The urgent need to find new effective and selective antitumor agents has found in nanotechnology a niche of opportunity and a growing field of research [1,2,3]. In this field, silver nanoparticles have been one of the most studied systems [4,5,6,7]. It has been identified that the physicochemical properties that modulate the antitumor activity of AgNPs are size, surface charge, and coating [2,8,9,10]. Many works have studied the effect of coating agent on selective cytotoxicity, identifying that the more stable the binding of the metallic surface with the coating agent, the lower its toxicity [8,11,12,13,14]. Polyvinylpyrrolidone (PVP) is one of the most used polymers to produce stable and effective AgNPs with remarkable antitumor effects [15,16,17,18]. However, we recently showed that the coating agent selection is not the only thing you need to worry about. The metallic content/coating agent ratio plays a significant role in the cytotoxic and genotoxic response of AgNPs [18,19,20,21].

Recently, many research articles proposed a minimal or negligible contribution of released silver ions to cytotoxicity and found that stable AgNP systems are primarily responsible for ROS overproduction within the exposed cells, which, in turn, trigger cell death pathways [19,20,22,23]. In the search for new treatment alternatives, during the last years, our research group has worked with a completely characterized PVP-AgNPs formulation, which exhibits in vitro [24] and in vivo [25] antimicrobial activity, virucidal effects [26,27,28], antiproliferative and antitumor activity [17,18], antiparasitic potential [29,30,31], immunomodulatory capacity [32,33], and others. We found that the amount of released Ag^+^ ions after 24 h incubation in the RPMI medium is lower than 1%. The [Metal]/[Coating agent] ratio used to synthesize Argovit^®^ provides this formulation with higher stability, responsible for the low Ag^+^ release [19]. The aforementioned substantially contributes to Argovit^®^ formulation specificity against pathogens and malignant cells with neither cytotoxic nor genotoxic damage on healthy cells [19,20]. By using another coating agent, in this case protein, but preserving the [Ag]/[coating agent] ratio used in the previous formulation, we found that the effectiveness as an antitumor and antiparasitic agent with high selectivity and little toxicity for BALB mice/c is similar [21].

Therefore, to confirm the role played by the [Ag]/[PVP] ratio on the antitumor activity, cytotoxic selectivity, and low toxicity, we studied five AgNP formulations with [Ag]/[PVP] ratios within a specific range. The cytotoxic activity of the AgNP formulations was evaluated against human colon tumor HCT-15 cells. Their selectivity was determined by exposing BALB/c mice primary cell cultures of the aorta, heart, liver, kidney, and spleen to different concentrations of the studied formulations. We assessed their median lethal dose (LD_50_) in BALB/c mice and performed histopathological analysis. Our results show that independently of the PVP employed, the amount of PVP is a crucial factor for the antitumor and wide therapeutic index (TI) of the AgNP formulations. All of them show significant cytotoxicity on human colon tumor cells triggered by ROS overproduction in mitochondria, promote a differential-cytotoxic effect on the tumor and non-transformed cells, and possess a wide therapeutic index with an LD_50_ that corresponds to Group 4 of the GSH classification. These findings open new opportunities for selective and safe nano-material formulation development for biomedical applications.

## 2. Methodology

### 2.1. AgNP Formulations

Five different AgNP formulations were kindly donated by the Scientific and Production Center Vector-Vita Ltd.^®^ (Novosibirsk, Russia). PVP-AgNP formulations are stable water suspensions with an overall concentration of 200 mg/mL. From here on, the formulations are labeled as AgNP1, AgNP2, AgNP3, AgNP4, and AgNP5. According to the manufacturer, differences between each AgNP formulation relies on the PVP used as coating agent or the synthesis conditions as follows: AgNP1: PVP K-15; AgNP2: PVP K-17; AgNP3: PVP K-17 with higher radiation potency used for the synthesis; AgNP4: PVP K-30, and AgNP5: PVP 12.6 ± 2.7 KDa. The synthetic procedures to obtain AgNP formulations are enclosed in the patents RU 2,602,534 C2 [34] and RU 2,602,741 C2 [35]. Molecular masses of PVP K-15, K-17, and K-30 PVP (Boai NKY Pharmaceuticals Ltd., Jiaozuo, China) are 8000–12,000 kDa, 10,000–16,000 kDa, and 45,0000–58,000 kDa, respectively. Based on the AgNP stock solution silver content, working solutions with final concentrations of 5.5 µM, 55.6 µM, 556 µM, and 5.5 mM were prepared with injectable water by serial dilutions. From here on, the concentrations used in this work indicate the metallic silver contained in the AgNP formulations.

### 2.2. Characterization

The morphology of the nanoparticles was analyzed by High Resolution Transmission Electron Microscopy (HR-TEM) using a JEM-2010 (JEOL^©^). AgNP average diameter (Ø_Ag_) and size distribution analysis was determined from TEM micrographs (n = 150) using ImageJ Software [36]. Zeta potential (ζ) and hydrodynamic diameter (Ø_hydro_) were determined by dynamic light scattering (DLS) using a Zetasizer Nano Ns DTS 1060 (Malvern Instruments^®^). Thermogravimetric analysis (TGA) and differential scanning calorimetry (DSC) measurements were performed with a DSC/TGA thermal analysis system (SDT Q600, Newtown, PA, USA) under argon atmosphere from 30 to 750 °C at a rate of 10 °C min^−1^. Moreover, AgNP dilutions were used to determine plasmon resonance by UV/Vis spectroscopy using a JENWAY 6705 spectrophotometer (Cole-Parmer^©^).

### 2.3. HCT-15 Cell Culture

HCT-15 human colorectal adenocarcinoma cell line was purchased from the American Type Culture Collection (ATCC^®^ CCL-225™, Manassas, VA, USA). HCT-15 cells were cultured in Petri dishes containing RPMI 1640 medium (Biowest^©^) supplemented with 10% fetal bovine serum (FBS; Biowest^©^ S1650, Mexico City, Mexico) and 0.01% antibiotic-antimycotic (Biowest^©^ L0010, streptomycin and penicillin G; Nuaillé, Maine-et-Loire, France). Cells were incubated at 37 °C in a 5% CO_2_ humidified atmosphere. The culture medium was refreshed every 2 days and cells were passaged at 80% confluency.

Cell viability was determined by seeding 20,000 cells/well in 96-well plates with 190 μL of supplemented culture media and exposed for 24 h to 10 µL of each AgNP formulation at final concentrations of 5.5 µM, 55.6 µM, 556 µM, and 5.5 mM. Treatments were added at the same time as cell seeding. Three independent experiments were performed by triplicate for each treatment. Untreated cells were used as negative control administered with 10 μL of complete culture medium. The reported IC_50_ for carboplatin (190 µM) [37], one of the first-line chemotherapeutic agents for human colon tumor treatment, was used as a positive control. Plates were incubated for 24 h at 37 °C in a 5% CO_2_ humidified atmosphere. Cell viability was determined using Vybrant™ CFDA SE cell tracer kit (Thermo Fisher Scientific, Waltham, MA, USA) by flow cytometry (Attune NxT flow cytometer, Thermo Fisher Scientific, Waltham, MA, USA).

### 2.4. Cell Death Modality

Cell death modality induced by exposure to AgNPs and carboplatin was determined using the Dead Cell Apoptosis Kit with Alexa Fluor^®^ 488 Annexin V (AV) and propidium iodide (PI) (Thermo Fisher, Waltham, MA, USA). After 24 h of incubation with the corresponding cytotoxic agent, the cells were harvested and washed with cold PBS and then resuspended in kit-provided binding buffer. The cells were then incubated with 5 μL of AV and 1 μL of PI solution at room temperature for 15 min. After incubation, cells were analyzed in the Attune NxT flow cytometer (Thermo Fisher Scientific, Waltham, MA, USA). At least 10,000 events/experiment were recorded with the BL1 channel for AV detection and VL3 for PI detection. Cells treated with hydrogen peroxide (H_2_O_2_) were considered as an apoptosis-positive control (positive for Annexin V; AV+); cells treated to break osmotic pressure were considered as a necrosis positive control (positive for propidium iodide; PI+).

### 2.5. Mitochondrial ROS Production

The mitochondrial superoxide (O_2_^−^) production was assessed using MitoSOX Red mitochondrial superoxide indicator (Invitrogen™, Waltham, MA, USA) by flow cytometry. Cells treated with H_2_O_2_ were employed as a positive control. After the exposure to AgNPs, 2 μL of MitoSOX reagent solution were added to each experiment and incubated for 10 min in the dark. Afterward, cells were analyzed in the Attune NxT flow cytometer (Thermo Fisher Scientific, Waltham, MA, USA). At least 10,000 events/experiment were recorded using the VL4 channel.

### 2.6. Primary Mouse Cell Cultures 

Primary mouse cultures were chosen to evaluate the cytotoxic effect of AgNP formulations in non-tumorigenic cells. Primary mouse cultures of aorta, heart, liver, kidney, and spleen were used in this work. For the latter, the viability of lymphocytes and monocytes was determined. The experimental procedure was performed with the approval of the Ethical Committee of the Autonomous University of Baja California (001/2018), and following the NOM-062-ZOO-1999. Briefly, BALB/c mice were euthanized by cervical dislocation and dissected. The cells were isolated from the organs by mechanical maceration, and tissue fragments were washed with phosphate buffer solution (PBS). After that, cells were centrifuged for 10 min at 2500 rpm. The supernatant was removed, and cells were washed with 100 μL of saturated saline solution followed by 900 μL of injectable water for erythrocyte lysis. Afterward, cells were washed three times and seeded in Petri dishes with RPMI 1640 culture medium supplemented with 10% *v*/*v* of fetal bovine serum (FBS, Biowest S1650, Mexico City, Mexico). Cells were incubated at 37 °C for 24 h. The cytotoxic effect was assessed by exposing the cells to 5.5 µM, 55.6 µM, 556 µM and 5.5 mM of AgNP formulations for 24 h. In the case of the spleen, the primary culture was incubated at 37 °C for at least 2 h to separate spleen monocytes from lymphocytes. After that time, monocytes adhere at the bottom of the dish allowing the collection of suspended lymphocytes. The lymphocytes collected were placed in a different container with fresh media. The same concentration ranges and exposure time used for the other primary cultures was employed to evaluate the cytotoxic effect on isolated lymphocytes and monocytes. After the incubation period, the cell type viability was determined independently by flow cytometry, based on cell size and cytoplasmic granularity. At least 10,000 events were analyzed using the Attune™ NxT flow cytometer (Thermo Fisher Scientific, Waltham, MA, USA).

### 2.7. Lethal Dose (LD_50_)

The lethal dose (LD_50_) of the five formulations was determined through the limit test according to OECD Guideline 420 for Acute Oral Toxicity Assay [38] using eight-week-old BALB/c mice with an average body weight range of 20–24 g. The experimental protocol was approved by the Ethical Committee of the Autonomous University of Baja California (001/2018), Mexico. Mice were assigned to polycarbonate cages and kept at 25 °C, 60% humidity, 12/12-h light-dark cycle with food and water *ad libitum*. Mice were orally administered 0.4 mL of the corresponding AgNPs every 2 h for a total of 12 doses in a period of 24 h. The total amount of silver administered to each mouse was in the range of 1067–1806 mg of metallic silver/Kg of body weight. Clinical signs, including skin and fur appearance, eyes, membranes, conduct, respiratory pattern, the presence of nausea, vomiting, diarrhea, tremors, convulsions, salivation, lethargy, sleep, coma, and death, were carefully registered throughout the experiment. Liver, kidney, spleen, lung, intestine, heart, and brain were removed and placed in 10% formaldehyde for histopathological evaluation.

### 2.8. Histopathology

The histopathologic analysis was performed as recommended by the OCDE Guide 420. Three to five-micrometer sections of paraffin-embedded organs stained with Hematoxylin and eosin (H&E) were used to identify possible tissue injuries.

### 2.9. Statistical Analysis

The obtained data were analyzed using GraphPad Prism version 9.0 software. The results were expressed as mean ± standard deviation of three experiments. Data were evaluated by two-way analysis of variance (ANOVA), followed by Tukey’s multiple comparison test. The results were considered statistically significant when *p* ˂ 0.05.

## 3. Results

### AgNPs Characterization

**Figure 1:**
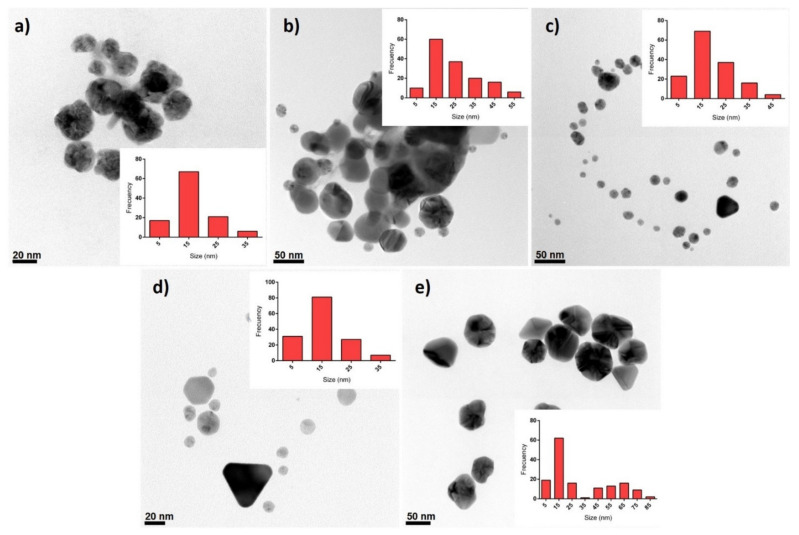
HR-TEM micrographs of the five AgNPs studied in this work (**a**) AgNP1, (**b**) AgNP2, (**c**) AgNP3, (**d**) AgNP4, and (**e**) AgNP5. The inset on each figure corresponds to the size histogram.

AgNP formulations were analyzed by TEM, UV-vis, DLS, and TGA. TEM micrographs revealed a spheroidal morphology of the metallic silver cores, although triangular shapes (possibly pyramidal prisms) were also observed (Figure 1). The average diameters (Ø_Ag_) of the metallic particles were 16.4 ± 8.0 nm, 25.4 ± 13.2 nm, 19.0 ± 9.3 nm, 16.4 ± 8.1 nm, 30.6 ± 23.2 nm for AgNP1 to AgNP5, respectively. Furthermore, size distributions showed that AgNP formulations were polydisperse with predominant diameters in the range of 10–20 nm.

**Figure 2:**
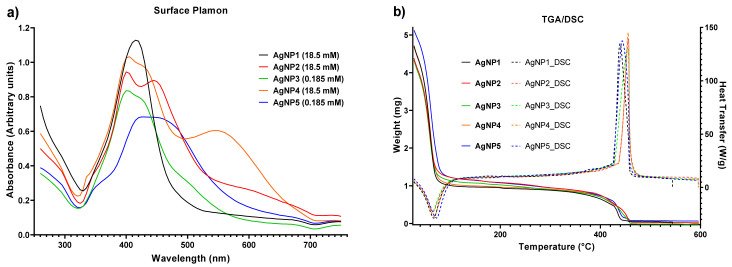
Surface plasmon resonance (**a**) and thermogravimetric analysis (TGA)/differential scanning calorimetry (DSC) (**b**) of the five formulations studied in this work. UV-vis spectra were acquired at different concentrations to assure the absorption values were close to unity. TGA and DSC analysis show the weight loss as a function of temperature and the amount of heat required to increase the temperature of the sample, respectively.

The UV-vis spectra of the five AgNPs formulations were acquired at different concentrations, as shown in Figure 2a. AgNP1 to AgNP4 showed an absorption peak between 400–415 nm. No defined peaks were observed for AgNP5, but a plateau-like distribution was seen. This absorption, common among formulations, corresponds to the characteristic surface plasmon resonance of nanometric silver [19]. Furthermore, AgNP2 and AgNP4 formulations showed a second peak of lower intensity. The apparition of two or more absorption peaks can be due to the presence of irregular particles (non-spherical) within the AgNP formulations, which results in the widening of the peak [39].

The hydrodynamic diameter (Ø_hydro_) and zeta potential (ζ) of the AgNP formulations are summarized in Table 1. A wide range of Ø_hydro_ was found; AgNP1 and AgNP4 showed the larger size with 448 and 483 nm, AgNP5 has a diameter of 121 nm, while diameters for AgNP2 and AgNP3 are 90 and 43 nm, respectively. The ζ potential values were within the range −5 to +5 mV, where the higher values (absolute values) correspond to the minor Ø_hydro_ registered. As the ζ values get close to zero, a higher Ø_hydro_ was found (Table 1).

The TGA/DSC analysis show the water, polymer, and metallic content for each formulation. The metallic content was in the range 1.1–1.3%, the protein coating was 19.6–24.5%, and water was 74.2–79.2% *w*/*w*. Those formulations with the highest coating protein content, AgNP2 and AgNP3, also showed the lower Ø_hydro_ values and the higher ζ potential values. These results highlight the role played by the coating on the physicochemical properties of these AgNP formulations.

## 4. Cell Viability

### 4.1. Antitumor Activity

**Figure 3:**
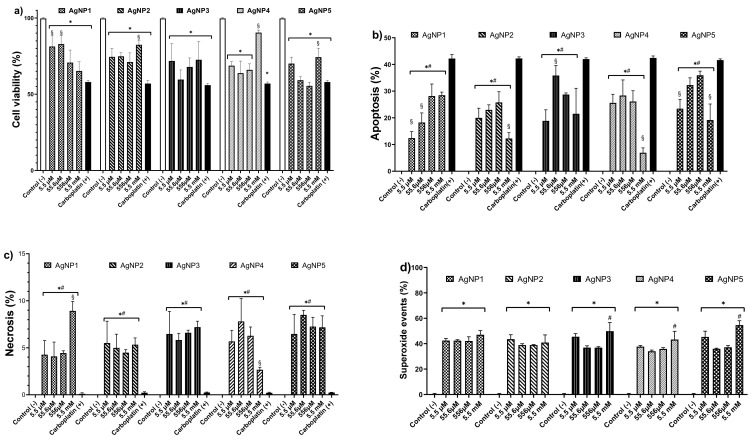
Cytotoxic effect on HCT-15 cultures after 24 h exposure to 5.5 µM, 55.6µM, 556µM and 5.5 mM of AgNP1–AgNP5. (**a**) Cell viability, (**b**) apoptosis, (**c**) necrosis, and (**d**) mitochondrial superoxide. * represents statistically significant differences (*p* < 0.05) compared with negative control, # represents statistically significant differences (*p* < 0.05) compared with carboplatin, and § represents statistically significant differences (*p* < 0.05) between the assessed concentrations of each AgNP formulation.

The cytotoxic effect of AgNPs on human colon tumor cells (HCT-15) was studied after 24 h exposure to concentrations from 5.5 µM to 5.5 mM of metallic silver contained in AgNP formulations (Figure 3). The five AgNP formulations evaluated in this work showed a similar cytotoxic effect on HCT-15 cultures to carboplatin (Figure 3a). From the lowest concentration of AgNP formulations assessed (5.5 µM), the observed cell viability decrease showed no significant statistical differences with carboplatin results, except for AgNP1, which reached the above-described effect at 556 µM. Formulations AgNP2–AgNP5 showed a decrease in their cytotoxicity at 5.5 mM, probably produced by nanoparticle aggregation (Figure 3a). In our experimental conditions, the reported IC_50_ for carboplatin on HCT-15 produced a cell viability decrease of 42–44% (Figure 3).

Interestingly, AgNP1 at concentrations from 5.5–556 µM showed a concentration-dependent increase in positive apoptotic events (Figure 3b). The other AgNP formulations always presented apoptosis within the range of 5–35%, regardless of the assessed concentration (Figure 3b). For necrosis, positive events were within the range of 2.5–7%, except for AgNP1, which was 9% at a concentration of 5.5 mM (Figure 3c).

Regardless of the concentration, all the formulations induced a 40% increase of mitochondrial superoxide in the cells exposed to AgNPs (Figure 3d). This mitochondrial ROS-overproduction could be the trigger of cell death pathways identified on HCT-15 cells (Figure 3b,c) exposed to AgNP1–AgNP5.

### 4.2. Primary Mouse Cultures

**Figure 4:**
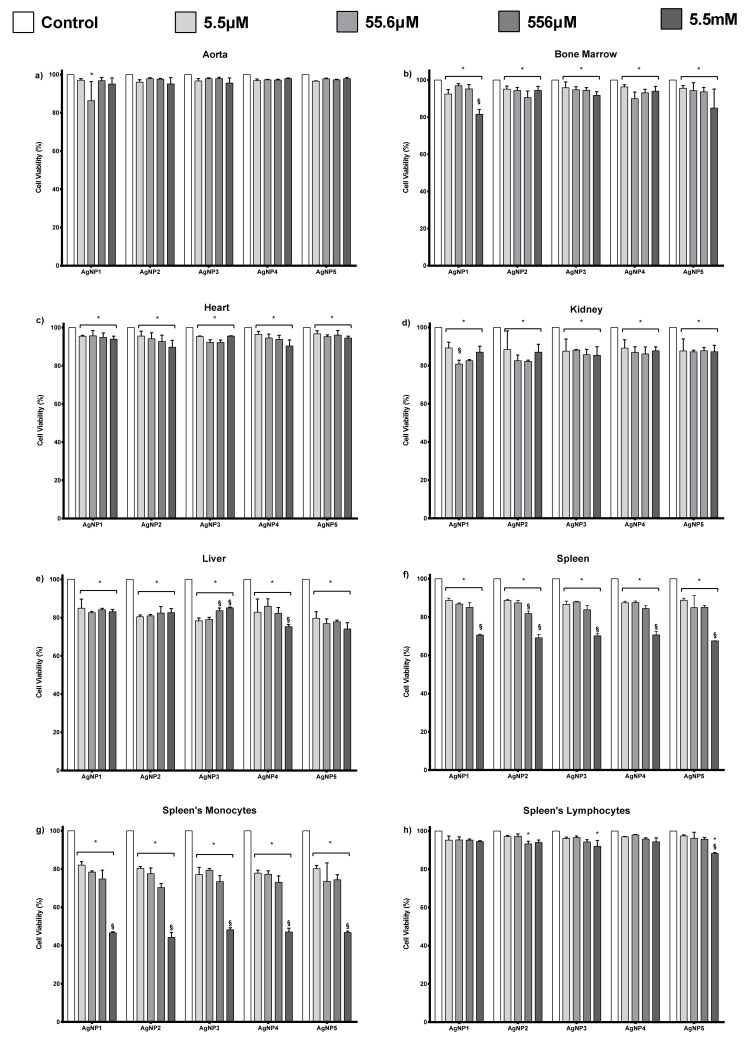
Cell viability of mouse primary culture exposed for 24 h to different concentrations (5.5 µM, 55.6 µM, 556 µM and 5.5 mM) of AgNP1–AgNP5 quantified by flow cytometry. (**a**) Aorta, (**b**) bone marrow, (**c**) heart, (**d**) kidney, (**e**) liver and (**f**) spleen. Items (**g**,**h**) show the cell viability of monocytes and lymphocytes obtained from the spleen; cell populations differentiated by size and granularity. * represents statistically significant differences (*p* < 0.05) compared with negative control and § represents statistically significant differences (*p* < 0.05) between the assessed concentrations of each AgNP formulation.

Cytotoxic selectivity of AgNP formulations AgNP1–AgNP5 was determined by exposing primary cultures of mouse aorta, bone marrow, heart, kidney, liver, and spleen to 5.5 µM, 55.6 µM, 556 µM, and 5.5 mM of the corresponding AgNP formulations. Similar to tumor cell cultures, the cell viability responses of each mice primary culture exposed to AgNPs are practically the same regardless of the concentration or formulation of AgNP assessed. The lowest effect on cell viability decrease was observed on the aorta cultures, where only a 5% decrease compared with the control was registered (Figure 4a). Bone marrow and heart primary cultures exposed to AgNPs presented a similar trend with a cell viability decrease of 5–10% (Figure 4b,c). Kidney and liver primary cultures showed a 15–20% diminishing cell viability after AgNP exposure (Figure 4d,e). Spleen cultures exposed to the highest concentration (5.5 mM) of the AgNP formulations assessed showed the most significant cell viability diminishing of all the series, a 30% decrease compared with the control (Figure 4f). Spleen lymphocytes exhibited a narrow reduction in cell viability, from 5–7% compared with the control (untreated cells, Figure 4h). On the other hand, spleen monocytes showed a meaningful decrease after exposure to 5.5 µM–556 µM of AgNPs (Figure 4g). Spleen monocytes’ cell viability abruptly dropped up to 46% when increasing concentration to 5.5 mM. The distinct difference in the cytotoxic effect between spleen lymphocytes and monocytes could be associated with their phagocytic capability.

### 4.3. Determination of Lethal Dose

The lethal dose of five AgNP formulations studied in this work were determined on BALB/c mice through a limit test. To avoid further suffering of experimental animals, we euthanized the surviving mice and concluded the study before 12 h of observation. All mice showed toxicity signs during the limit tests; however, the administered dose of silver contained on the AgNP formulation is considerably high, as shown in Table 2. The median lethal doses determined for each formulation are compiled in Table 2.

### 4.4. Histological Analysis

**Figure 5:**
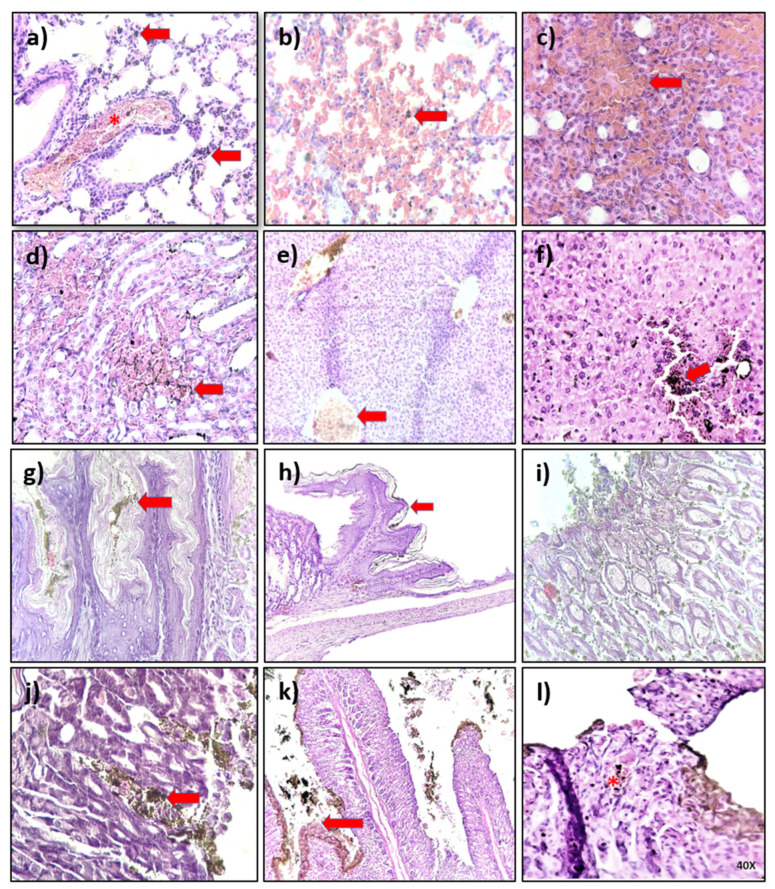
Histological analysis of mice organs exposed to AgNPs formulations AgNP1–AgNP5. The photographs are representative of the damage, when observed, on the lung (**a**,**b**); kidney (**c**,**d**); liver (**e**,**f**); esophagus (**g**); gastroesophageal union (**h**); stomach (**i**,**j**), and intestine (**k**,**l**). Red arrows indicate accumulation of dark non-cellular material. Asterisks show cellular damage.

The histological analysis of organs from mice exposed to AgNP formulations during the limit test showed minimal toxic effects after the observational period. Table 3 compiles the most relevant findings in the lung, kidney, heart, spleen, liver, esophagus, stomach, and intestine; however, it is imperative to note that described damage is only observed in some exposed mice. In general, images show the accumulation of dark non-cellular material (probably AgNPs) in different tissues’ interstitium. The most evident accumulation is observed in the gastrointestinal system (esophagus (Figure 5g), the gastroesophageal union (Figure 5h), stomach (Figure 5i), small intestine (Figure 5k,l), and the liver (Figure 5f)). The observed accumulation on these organs could be associated with the administration route. Despite the accumulation, no tissue damage was observed within 12 h of the acute toxicity study. These results agree with those observed in the in vitro cytotoxic assays using primary mouse cultures. AgNP formulations showed the most significant cytotoxic effects on the organ cells that showed the highest accumulation on the histological observations.

## 5. Discussion

In this study, we evaluated the cytotoxicity of five new AgNP formulations against human colon tumor HCT-15 cells. The AgNP formulations have a [Ag]/[PVP] ratio within the range [1.19–1.32% *w*/*w*]/[19.6–24.5% *w*/*w*] as shown in Table 1 (or [5–5.7% *w*/*w*]/[94.3–95% *w*/*w*] if the dry formulation is considered). The shape of the nanoparticles is predominantly spherical with a size distribution within the range of 5–80 nm (Table 1). Interestingly, both formulations with lowest PVP content, AgNP1 and AgNP4, exhibited a higher hydrodynamic diameter and a lower surface charge (Table 1). These results agree with those obtained by Kyrychenko and co-workers with molecular dynamics studies. They found that an increase in the amount and the length of PVP oligomers increased the coating coverage and avoided AgNP self-aggregation [40].

Several AgNPs that use PVP as a coating agent minimized cytotoxic and genotoxic effects [8,41,42], but only significantly increasing the amount of PVP leads to an absence of toxicity, as we previously reported in several in vitro and in vivo models [18,19,20,33]. Several AgNPs promote ROS overproduction, identified as one of the apoptosis triggers in tumor cells exposed to them. However, many exhibited genotoxic damage even when PVP was used as the coating agent [11,15,43,44,45,46].

In the case of AgNP formulations evaluated in human colon tumor cells, most of them present their cytotoxic effect by ROS overproduction, which leads to apoptosis induction; however, limited information about coating agent or formulation stability is available [47,48,49,50,51,52,53,54,55,56,57]. The results of this work agree with the general trend, all the formulations assessed promote mitochondrial ROS overproduction (Figure 3d) and trigger apoptosis (Figure 3b) as the major cell death pathway. AgNP’s mitochondrial ROS overproduction could be associated with is capacity to modulate the expression of NOX4, the gene that encodes the enzyme NADPH 4 oxidase in humans. Lee and co-workers found that human colon tumor cells HCT116 exposed to AgNPs show mitochondrial disfunction (MD) and endoplasmic reticulum (ER) stress associated with NOX4 expression. However, induced damage was significantly inhibited by pretreatment with diphenyleneiodonium (DPI) or 4-phenyl butyric acid (4-PBA), specific inhibitors of NADPH 4 oxidase and ER stress, respectively [58].

The necrosis observed (Figure 3c) could be associated with the high cytotoxic potency of the AgNP formulations, which in general are more than 30 times more potent than carboplatin. Cell viability observed in cells exposed to 190 µM of carboplatin is similar to that observed in cells exposed to 5.5 µM of AgNP2–AgNP5. Necrosis could be decreased or avoided with frequent administration of a lower concentration. The cytotoxicity of the five formulations exhibits a non-concentration dependent effect. This behavior was already observed in human colorectal cancer (DLD-1 and HT29) [17] and murine melanoma (B16F10) [18] exposed to a PVP-AgNP formulation with a similar composition (Ag 1.2% *w*/*w* and PVP 18.8% *w*/*w*). Interestingly, the aforementioned formulation induces a concentration-dependent cytotoxic activity on human breast cancer (MDA-MB-231 and MCF-7), cervix (HeLa), prostate (DU-145), and lung (H1299 and H1437). The above shows that formulations with this type of [metallic content]/[coating agent] ratio present selectivity in cytotoxic activity according to the cancer cell type.

The selective cytotoxic effect was also observed in primary mouse cultures. Liver, kidney, and spleen cells are most sensitive to AgNPs, as shown in Figure 4. The higher sensitivity of these cells compared to the aorta, bone marrow, and heart cultures are related to their enhanced phagocytic capabilities to incorporate AgNPs, also favored by the tendency of AgNPs to accumulate in the liver regardless of the administration route [7,59,60,61,62,63]. Enhanced cytotoxicity on phagocytic cells was also observed on spleen cultures, where a pronounced cell viability decrease was observed on monocytes without significant modification of lymphocyte viability (Figure 4g,h). Despite the cytotoxic effect registered in vitro on liver, kidney, and spleen cultures, no evidence of tissue damage was observed on the histological analysis after oral administration (Figure 5) used to determine the medium lethal dose (LD_50_) through a limit test.

Histological analysis showed the accumulation of AgNPs, mainly in the gastrointestinal system. Interestingly, even with the significant accumulation in the tissue, minimal damage was observed. The lack of damage could be attributed to the considerable amount of coating agent, enhanced by its high clearance rate, as we have shown in previous works [18,21,64,65,66,67]. These features definitely contribute to the wide therapeutic window found for the five formulations assessed in this work. They all present an LD_50_ value within the range of 1067–1548 mg of silver/Kg of mouse body weight. The LD_50_ values found place the five formulations in category 4 of the GSH classification, the same category as carboplatin (343 mg/Kg by oral administration) [68]. Considering AgNPs and carboplatin cytotoxic effects on HCT-15 and their LD_50_ values, a rough value of therapeutic index (TI) can be obtained by LD_50_/IC_50_, resulting in TI values within the range of 179–304 for AgNP formulations and 4.86 for carboplatin. In summary, the results obtained in this work show that a high amount of PVP used as a coating agent to obtain AgNP formulations, independently of polymer length, generates a wide therapeutic window for selective cytotoxic agents to BALB/c mice compared to the first-line chemotherapeutic agent, carboplatin.

## 6. Conclusions

In this work, we present the complete characterization of five PVP-AgNP formulations with composition within the range Ag: 1.14–1.32% *w*/*w*, PVP: 19.6–24.5%, and H_2_O: 74.2–79.2%. The AgNPs have spherical shapes with an average size range of 16–30 nm determined by TEM. All formulations exhibit a potency 34.5 times higher than carboplatin against human colorectal HCT-15 cells, except for AgNP1, which presented twice the cytotoxic potency of the platin metallodrug. The studied formulations show high cytotoxic selectivity and enhanced sensitivity for phagocytic cells. Despite the in vitro cell viability decrease found, a remarkably wide therapeutic window on BALB/c mice was observed. Histopathological analysis showed the accumulation of non-cellular material, mainly in the gastrointestinal system, without significant damage to exposed tissue. The selective cytotoxic effect and minimal damage observed on the mice tissue after oral administration of the five AgNP formulations are associated with the [metal]/[coating agent] ratio used to produce them. This feature placed all of the formulations at Category 4 of the Globally Harmonized System of Classification and Labelling of Chemicals. Our results provide an additional example of the rational design of safe nanomaterials with selective cytotoxic potency.

## Figures and Tables

**Table 1:** Physicochemical properties for the five AgNP formulations studied in this work.

Properties	AgNP1	AgNP2	AgNP3	AgNP4	AgNP5
Average diameter of metallic cores Ø_Ag_ (nm)	16.4 ± 8.0	25.4 ± 13.2	19.0 ± 9.3	16.4 ± 8.1	30.6 ± 23.2
TEM size distribution (nm)	5–40	5–60	5–40	5–40	5–80
PVP	K-15 ^a^	K-17 ^b^	K-17 ^b^	K-30 ^c^	12.6 KDa
Hydrodynamic diameter Ø_hydro_ (nm)	448.7	90.4	43.8	483.2	121.1
Polydispersity index (PDI)	0.813	0.270	0.433	0.555	0.280
Zeta potential ζ (mV)	−0.872	−4.56	−5.13	−0.464	−1.46
Surface plasmon resonance (λ)	415	402, 444	402	406, 549	429
TGA analysis					
Metallic silver (% *w*/*w*)	1.14 ± 0.02	1.32 ± 0.05	1.26 ± 0.03	1.19 ± 0.01	1.31± 0.01
PVP (% *w*/*w*)	19.62 ± 0.30	24.49 ± 0.70	24.43 ± 0.20	20.92 ± 0.42	21.67 ± 0.50
H_2_O (% *w*/*w*)	79.24 ± 0.45	74.25 ± 0.60	75.74 ± 0.25	77.89 ± 0.80	77.02 ± 0.40
Morphology	Spherical	Spherical	Mostly Spherical	Mostly Spherical	Spherical

^a^ K-15: 8000–12,000 kDa; ^b^ K-17: 10,000–16,000 kDa; ^c^ K-30: 45,000–58,000 kDa.

**Table 2:** Toxicity signs and median lethal dose (LD_50_) of AgNP formulations assessed in this work.

Formulation	Toxicity Sings ^1^	Lethal Dose (mg/Kg) ^2^	GHS Category ^3^
AgNP1	Slouching, lethargy, hypothermia, dehydration, over-reactive to tactile stimulation.	1067	4
AgNP2	Slouching, ruffed hair, vomiting attempt, lethargy, dyspnea, diarrhea, prostration, hypothermia, and seizure.	1067	4
AgNP3	Lethargy, ruffed hair, slouching, and diarrhea.	1290	4
AgNP4	Lethargy, slouching, and ruffed hair.	1806	4
AgNP5	Lethargy, slouching, ruffed hair, and closed eyes.	1548	4

^1^ This column shows all the toxicity signs observed with each formulation. ^2^ The mean lethal dose (LD_50_) of silver contained on each AgNPs formulation per kilogram of mouse body weight. ^3^ Classification based on the Globally Harmonized System (GHS) of Classification and Labeling of Chemicals.

**Table 3:** Histological findings in organs from mice exposed to AgNP1–AgNP5.

	AgNP1	AgNP2	AgNP3	AgNP4	AgNP5
**Lung**	Congestion	Congestion hemorrhage	Congestion hemorrhage	Congestion hemorrhage	Congestion hemorrhage
**Kidney**	Focal hemorrhage	Focal hemorrhage	Focal hemorrhage	Focal hemorrhage	Focal hemorrhage
**Heart 1**	Congestion	Congestion	Congestion	Congestion	Congestion
**Spleen 1**	Congestion	Congestion	Congestion	Congestion	Congestion
**Liver 1**	Congestion	Congestion	Congestion	Congestion	Congestion
**Esophagus 1**	Material deposit on the mucosa	Material deposit on the mucosa	Material deposit on the mucosa	Material deposit on the mucosa	Material deposit on the mucosa
**Stomach 1**	Epithelial damage	Epithelial damage	Epithelial damage	Epithelial damage	Epithelial damage
**Intestine 1**	Material deposit on the mucosa	Material deposit on the mucosa	Material deposit on the mucosa	Material deposit on the mucosa	Material deposit on the mucosa

^1^ When observed, damage described is diffuse.

## Data Availability

Not applicable.
